# Effect of continuous nursing interventions on valve noise-related anxiety and quality of life in patients who underwent mechanical mitral valve replacement

**DOI:** 10.1186/s13019-020-01117-y

**Published:** 2020-05-06

**Authors:** Qiu-Yu Chen, Ning Xu, Shu-Ting Huang, Ze-Wei Lin, Hua Cao, Liang-Wan Chen, Qiang Chen

**Affiliations:** 1 Department of Cardiac Surgery, Fujian Maternity and Child Health Hospital, Affiliated Hospital of Fujian Medical University, Fuzhou, China; 2 Department of Cardiovascular Surgery, Union Hospital, Fujian Medical University, Fuzhou, People’s Republic of China

**Keywords:** Nursing interventions, Anxiety, Quality of life, Mechanical valve replacement

## Abstract

**Objective:**

The purpose of this study was to evaluate the effect of continuous nursing interventions on valve noise-related anxiety in patients undergoing mechanical mitral valve replacement (MVR) and to analyze its impact on patient quality of life.

**Methods:**

Ninety patients who underwent mechanical MVR were divided into two groups. All patients in group A received routine nursing care. In addition to this intervention, an assigned nurse periodically provided patients in group B with continuous nursing interventions and ongoing health consultations during a 1-year follow-up. A hospital anxiety and depression (HAD) scale, a customized questionnaire and a Short Form Health Status 36 (SF-36) score questionnaire were used as the research tools.

**Results:**

The postoperative HAD scores were better in group B than in group A, but the differences in most of the data were not statistically significant between the groups, except for HA sections 0–7 and 11–21. Based on the customized questionnaire, the subjective disturbance level was significantly lower in group B than in group A (the results of Q1 and Q4 were statistically significant). With regard to the SF-36 scores, group B was superior to group A in general health, emotional function and mental health, while the other dimensions had no significant difference.

**Conclusions:**

Compared with routine care, patients who received continuous care intervention after mechanical MVR had fewer anxiety symptoms and better quality of life.

## Background

In the population of long-term survivors after mechanical mitral valve replacement (MVR), uninterrupted “click” sounds of the mechanical valve may plague patients in a manner similar to that of surgical complications after mechanical MVR. This may lead to anxiety and psychological stress and, in turn, affect patient quality of life [1, 2]. Studies that evaluate the impact of mechanical valve noise on quality of life are mostly available in Western countries [3–5]. However, there is still a high incidence of rheumatic heart disease in developing countries, especially in China, and there are a large number of patients who survive for a long time after mechanical MVR. To date, no studies have investigated whether continuous nursing interventions and related ongoing health consultations affect the anxiety symptoms associated with mechanical mitral valve noise, which affects patient quality of life. Therefore, the aim of this study was to determine the effects of continuous nursing interventions on postoperative mechanical valve-related anxiety symptoms and postoperative quality of life.

## Materials and methods

### Calculation of the study sample size

According to the results of the presurvey, the alpha value was set to 0.05, and the power was set to 0.90. The minimum sample size was calculated to be 41 patients in each group. Considering a 10% rate of possible loss to follow-up, the total sample size was set as 90 patients.

### Patients

This study was approved by the Ethics Committee of Fujian Medical University, China, and written informed consent was obtained from all patients before participating in the study. Patients who underwent mechanical MVR in our center between December 2017 and December 2018 were included in the study. The SJM mechanical mitral valve (25–29#) and intra-annular implantation technique were used in all patients (St. Jude Medical, Minnesota, USA). The following inclusion criteria were used: simple mechanical MVR, postoperative cardiac function level I, normal anatomical structure and ejection fraction of the heart, and heart rate 60–100 beats/min. The exclusion criteria were as follows: patients who underwent secondary surgery, preoperative heart failure, malignant arrhythmia accompanied by other cardiac malformations (including aortic valve lesions, left atrial thrombus, giant left atrium, coronary heart disease, cardiomyopathy), severe liver and kidney dysfunction and other organ dysfunction, patients with a disturbance of consciousness or mental illness, communication disorders and speech disorders, patients with poor compliance or incomplete data and patients older than 70 years of age.

The researchers randomly divided eligible parents into the routine nursing care group (group A) and the continuous care intervention group (group B) based on computer-generated random numbers (0 or 1). The patients who generated 0 were assigned to group A (*n* = 45), and the patients who generated 1 were assigned to group B (n = 45). The patients in group B received the same nursing intervention as group A in addition to an assigned nurse who periodically provided patients with continuous nursing interventions and ongoing health consultations before and after surgery during a 1-year follow-up. Mechanical mitral valve sounds were measured by a high-end professional hand-held sound level meter (Tecman sound level meter, which was made by Hong Kong Tekman Electronic Instrument Co., LTD., model TM 834). All patients were asked to complete a hospital anxiety and depression (HAD) scale before participating in the study, and valve sounds were directly measured before discharge. The relative preoperative data are shown in Table 1. One year after surgery, the patients were reevaluated in outpatient clinics. In addition to a physical examination, cardiac echocardiography, electrocardiography and the relevant examination of anticoagulation, valve sounds were directly measured again, and all patients were asked to complete a HAD scale, a customized questionnaire and a Short Form Health Status 36 (SF-36) score questionnaire. Specialized volunteers helped guide the participants in answering the questions if necessary, but they did not interfere with the patients’ choices. Another researcher was responsible for the collection and organization of data.

**Table 1:** Clinical data comparison between two groups of patients

Item	Group A	Group B	***p*** value
**N**	45	45	
**Gender(M/F)**	21/24	23/22	0.673
**Age (years)**	62.6 ± 5.9	62.4 ± 5.0	0.115
**Weight (kg)**	64.0 ± 4.6	66.1 ± 4.1	0.634
**Concomitant hypertension**	31 (68.9%)	33 (73.3%)	0.642
**Concomitant diabetes mellitus**	29 (64.4%)	26 (57.8%)	0.516
**Smoking history**	18 (40.0%)	21 (46.7%)	0.523
**The sound of valve at discharge**	64.6 ± 7.4 dB	65.2 ± 9.2 dB	0.701
**The sound of valve during the 1 year’s follow up**	63.9 ± 6.1 dB	64.4 ± 7.9 dB	0.625

### Nursing education

Both groups of patients received routine nursing care 1 to 2 days prior to surgery. The content was provided by a nurse in the ward during a brief visit and included general issues related to surgery, duration of the operation, postoperative pain management, postoperative environment in the intensive care unit and spontaneous stress, expected stay in the intensive care unit, knowledge of endotracheal intubation, potential postoperative complications, postoperative long-term pain management, lung care, nutrition needs, anticoagulation and rehabilitation. In addition to the above intervention, the assigned nurse also periodically provided patients in group B with continuous nursing interventions and ongoing health consultations during a 1-year follow-up. The content consisted of 3 main aspects [1]: information regarding the pathophysiology of valvular disease and the necessity of MVR [2], the mechanical valve material, the mechanical noise generated by closure of the mechanical valve and disturbance caused by the noise, highlighting that there is no effect of valve noise on hemodynamics or physical condition, and how to overcome the interference of noise in daily life, and [3] ongoing healthy and psychological counseling and intervention to guide patients to complete anticoagulation therapy, a healthy recovery, and a return to normal social life as soon as hemodynamics improved after valve surgery. After discharge, such interventions were continued via outpatient visits, telephone calls, and social apps (WeChat platform).

### HAD scale

The HAD scale is used to assess the status of depression and anxiety, and each entry is divided into 4 levels [6, 7]. A total score of 0–7 represents no depression or anxiety, a score of 8–10 represents possible or “threshold” depression or anxiety, and a total score of 11–20 represents significant depression or anxiety.

### Customized questionnaire

A customized questionnaire was designed to evaluate the impact of mechanical mitral valve noise on patient quality of life. In this customized questionnaire, the following items were included, and the answer was either yes or no:

Q1: Can the noise from your heart valve be heard without intention?

Q2: Can you hear sounds from your heart valve in noisy environments?

Q3: Can people around you hear sounds from your heart valve?

Q4: Do the sounds from your heart valve interrupt your sleep?

Q5: Do the sounds from your heart valve bother you during daily life and work?

Q6: Are the sounds from your heart valve intolerable, causing mental stress?

Q7: Are people around you unable to tolerate the sounds from your heart valve?

### SF-36 score

The standard SF-36 score questionnaire was used in this study, which includes eight scales: physical functioning (I), social functioning (II), role-physical (III), role-emotional (IV), mental health (V), vitality (VI), bodily pain (VII) and general health (VIII) [8, 9]. For practical purposes, a Chinese translated version of the standard SF-36 questionnaire was used.

### Statistical analysis

SPSS 25.0 software was used for statistical analysis. Measured data were expressed as the average ± standard deviation. All the data were tested for normality: the t-test was used to compare the normally distributed data, and the rank-sum test was used to compare the nonnormally distributed data. For the clinical baseline data, a t-test was used to compare sex, age and weight, and a chi-square test was used to compare concomitant hypertension, diabetes mellitus, smoking history, and HAD scale scores. The Mann-Whitney test was used to compare the sound of the valve between the two groups. During the 1-year follow-up, the chi-square test was used to compare the HAD scale scores and customized questionnaire scores of the two groups. The SF-36 scores of the two groups were compared by the Mann-Whitney test. *P* < 0.05 was considered statistically significant.

## Results

There were no significant differences in general data between the 2 groups (Table 1). There were also no significant differences in the direct measurement of the sound of the mechanical valve at discharge or during the 1-year follow-up. Regarding the HAD scores, there were also no significant differences between the 2 groups before participating in the study. The postoperative HAD scores in group B were better than those in group A, but the differences in most of the data were not statistically significant, except for HA sections 0–7 and 11–21, which were significantly different between the groups. Compared with the preparticipation data, the HAD scores during the 1-year follow-up were significantly worse in certain score intervals (Tables 2). In the comparison of the customized questionnaires, the subjective disturbance level was significantly lower in group B than in group A. The results of the first and fourth questions were significantly different between these two groups (Q1: group A 28/45 vs. group B 9/45; Q4: group A 27/45 vs. group B 11/45). In addition, 7 patients in group B felt that the sounds from the heart valve were unbearable, causing mental stress, while in group A, 9 patients felt the same. However, there was no significant difference between the groups (Table 3). The SF-36 score revealed statistically significant differences in general health, emotional function and mental health but not in other domains (Table 4).

**Table 2:** The comparative data of HAD scale between two groups of patients

Item	Group A	Group B	p value
**HA (before participating in the study)**			
**0–7**	36 (80.0%)	35 (77.8%)	0.79
**8–10**	7 (15.6%)	9 (20.0%)	0.58
**11–21**	2 (4.4%)	1 (2.2%)	0.55
**HA (during 1 year’s follow up)**			
**0–7**	12 (26.7%)	26 (57.7%)	0.002
**8–10**	16 (35.5%)	14 (31.1%)	0.65
**11–21**	17 (37.8%)	5 (11.2%)	0.003
**p**	0.000	0.07	
**HD (before participating in the study)**			
**0–7**	34 (75.6%)	37 (82.3%)	0.43
**8–10**	9 (20.0%)	6 (13.3%)	0.39
**11–21**	2 (4.4%)	2 (4.4%)	1
**HD (during 1 year’s follow up)**			
**0–7**	16 (35.5%)	20 (44.4%)	0.39
**8–10**	14 (31.1%)	16 (35.5%)	0.65
**11–21**	15 (33.4%)	9 (20.1%)	0.15
**p**	0.0001	0.0008

**Table 3:** The comparative data of customized questionnaire between two groups of patients at 1 year after surgery

Item	Group A	Group B	p value
Q1	28 (62.2%)	9 (20.0%)	0.000
Q2	19 (42.2%)	15 (33.3%)	0.38
Q3	21 (46.7%)	17 (37.7%)	0.39
Q4	27 (60.0%)	11 (24.4%)	0.000
Q5	19 (42.2%)	18 (40%)	0.83
Q6	14 (31.1%)	12 (26.7%)	0.64
Q7	9 (20.0%)	7 (15.6%)	0.58

**Table 4:** The comparative data of SF-36 scale between two groups of patients

Item	Group A	Group B	p value
**PF**	57.7 ± 12.4	56 ± 8.2	0.634
**RP**	59.4 ± 21.5	58.3 ± 21.9	0.807
**BP**	53.8 ± 10.6	53.2 ± 15.8	0.935
**GH**	54.2 ± 12.5	50.3 ± 14.4	0.005
**VT**	54.4 ± 11.2	54.8 ± 11.7	0.883
**SF**	56.7 ± 14.5	59.9 ± 16.5	0.474
**RE**	43.7 ± 19.9	77.0 ± 17.1	0.000
**MH**	73.5 ± 6.4	77.9 ± 2.9	0.000
**HT**	54.4 ± 17.0	56.6 ± 18.7	0.870

## Discussion

Mechanical heart valve replacement is one of the most common procedures in cardiac surgery and is more suitable for patients with heart valve disease than those without. A large number of patients undergo mechanical MVR every year and experience long-term survival [10–12]. Mechanical valve-related noise is inevitable in patients who undergo mechanical valve replacement rather than biological valve replacement [13]. The noise generated by the closure of the mechanical mitral valve may lead to postoperative anxiety and psychological stress and affect patient quality of life. Increasingly more researchers are concerned that the noise generated by mechanical valves may negatively affect the quality of life of patients. Studies have shown that at least one-third of patients after mechanical heart valve replacement are concerned about the sound from the mechanical valves and believe that the sounds will affect their quality of life, although few complain that the sounds seriously affect survival [14–16]. A case report even described a patient who underwent secondary surgery for bioprosthetic valve replacement due to noise intolerance to mechanical valve closure [17]. Moreover, patients who undergo mechanical MVR may experience pain in the chest, and the recovery of cardiopulmonary function usually takes half a year to 1 year. Noise leads to negative emotions such as anxiety and depression in the early postoperative period and is not conducive to prognosis and recovery. Therefore, it is of great clinical significance for nurses to provide preoperative health education and postoperative psychological counseling for patients who undergo mechanical MVR. At present, there are few studies on continuous nursing interventions on mechanical valve noise-related postoperative anxiety and quality of life. We designed this study to investigate this issue to guide clinical work.

In this study, the uniform type mechanical valve sounds of the two groups were directly measured before discharge and at the 1-year follow-up. The results objectively showed no difference in valve noise between the two groups, except for the influence of real valve noise. The impact of valve noise on postoperative anxiety symptoms and quality of life was evaluated by using the HAD scale, a customized questionnaire, and the SF-36 questionnaire. Uninterrupted valve noise may cause anxiety or depression, which could lead to a serious disruption to the patient’s daily life, especially for those who are highly sensitive. The postoperative HAD scores were better in group B than in group A, especially for HA sections 0–7 and 11–21, which were significantly different between groups. We concluded that the patients in group B who received continuous professional nursing interventions and psychological guidance had fewer anxiety symptoms than those who did not. This was due to the one-on-one communication guidance according to the patient’s individual situation. The nurse provided detailed and individualized information to improve patient awareness of the disease and the operation. These practices can ensure that patients understand the postoperative noise made by mechanical valves and are prepared to address this issue. Furthermore, these interactions with nurses can improve the trust patients have with medical staff and thereby alleviate negative emotions of anxiety and depression that begin during hospitalization and after discharge from the hospital.

According to the results of the postoperative questionnaires, the patients in group B felt less subjective disturbance than did those in group A. This applies not only to the general judgment of patients but also to the degree of disturbance in specific situations (for example, the valve noise cannot be heard during sleep or without intention). Specialized guidance regarding valve noise before surgery can ensure patients understand and are able to manage the impact of mechanical valve noise on psychological and living aspects during postoperative recovery. The SF-36 questionnaire is widely recognized and applied as one of the most important scales for health-related quality of life assessment. In cardiac surgery and related fields, it has been reported in the literature that the SF-36 questionnaire is suitable for assessing the health-related quality of life of patients with heart disease. We found that few studies have used the SF-36 questionnaire to evaluate the quality of life of patients after mechanical MVR. The SF-36 scores were higher in group B than in group A concerning general health, emotional function and mental health. According to these results, we believe that preoperative continuous nursing interventions have a significant effect on patients after mechanical MVR and can improve quality of life.

Clearly, valve noise is associated with postoperative anxiety symptoms and quality of life. We studied the effects of continuous nursing interventions on valve noise-related anxiety symptoms and quality of life after mechanical MVR. However, this study did not include factors such as cultural status, economic income, hospitalization time, overall cost, etc. Relatively speaking, the subjective factors of the tools used in this study have more weight. In addition, this study was not a clinical double-blind controlled trial. Thus, there is a certain bias in the data collected from the patients and their family members. In the future, studies with larger sample sizes and longer follow-up periods should be performed to validate these conclusions.

## Conclusion

In summary, continuous nursing interventions and ongoing health consultations can alleviate anxiety and depression caused by valve noise in patients with mechanical MVR and improve quality of life and, thus, are worthy of clinical promotion and application.

## Data Availability

Data sharing not applicable to this article as no data sets were generated or analyzed during the current study.

## Abbreviations

HAD: Hospital anxiety and depression
SF-36: Short Form Health Status
